# Design and Behavior of Lightweight Flexible Structure with Spatial Pattern Reducing Contact Surface Fraction

**DOI:** 10.3390/polym15193896

**Published:** 2023-09-26

**Authors:** David Rybansky, Pavel Marsalek, Martin Sotola, Juraj Hroncek, Lukas Drahorad, Ondrej Kusnir, Jiri Prokop

**Affiliations:** 1 Department of Applied Mechanics, Faculty of Mechanical Engineering, VSB—Technical University of Ostrava, 17. listopadu 2172/15, 708 00 Ostrava, Czech Republic; 2 Department of Surgical Studies, Faculty of Medicine, University of Ostrava, Dvorakova 7, 701 03 Ostrava, Czech Republic; 3 Department of Surgery, University Hospital Ostrava, 17. listopadu 1790/5, 708 00 Ostrava, Czech Republic

**Keywords:** flexible, lightweight, spatial, structure, stiffness, biomedical, PA12, SLS

## Abstract

Flexible structures are increasingly important in biomedical applications, where they can be used to achieve adaptable designs. This paper presents a study of the design and behavior of 3D-printed lightweight flexible structures. In this work, we focus on the design principles and numerical modelling of spatial patterns, as well as their mechanical properties and behavior under various loads. Contact surface fraction was determined as the ratio of the surface area of the printed pattern to the surface area of the entire curved surface. The objective of this work is to design a spatial pattern reducing contact surface fraction and develop a non-linear numerical model evaluating the structure’s stiffness; in addition, we aimed to identify the best design pattern with respect to its stiffness:mass ratio. The experimental verification of the numerical model is performed on 3D-printed prototypes prepared using the Selective Laser Sintering (SLS) method and made of Nylon—Polyamide 12. The obtained results provide insights into designing and optimizing lightweight external biomedical applications such as prostheses, orthoses, helmets, or adaptive cushions.

## 1. Introduction

Medical and protective aids are currently widely used means of treatment, pain reduction, or health protection. In most cases, these aids are mass-produced with uniform properties, which may not suit all users. An alternative way is to produce these aids, such as shoe insoles, cranial orthosis, protective helmets and shoes for elite athletes, etc., with individualized shapes and properties so that they serve their purpose as well as possible. However, the possibility of such use depends on a number of factors such as mechanical properties (weight and stiffness), manufacturability, appearance, etc. Analysis of these and other factors has been reviewed by Seharing et al. [1].

The procedure using additive manufacturing (AM) can be used to design personalized aids with unique properties for each user. The utilization of 3D printing in orthotic inserts design to reduce foot pain was presented by Sharma et al. [2]. Determination of the mechanical properties of the foot orthosis and their influence on its biomechanical effect on individuals with flexible flatfoot was presented in the paper [3] by Lin et al. These orthoses were 3D-printed with different orientations and the maximum compressive load and stiffness were calculated based on experimental testing. To use AM for breast support purposes, Prokop et al. dealt with determining breast stiffness in the paper [4]. Holmes et al. presented a study of the mechanical behavior of 3D printed gyroid structures, which can be used for customized cushions to prevent pressure ulcers [5]. Sotola et al. presented a novel approach to the design of a transtibial prosthesis bed stump using topology optimization and 3D printing [6] and they also presented a sensitivity analysis of key formulations of topology optimization [7]. AM products’ geometry and mechanical properties depend on technological parameters. Kundera et al.analyzed the influence of these AM parameters, especially Selective Laser Sintering (SLS), on the quality of the resulting geometric surface texture [8]. They also studied mechanical properties of models made of polyamide PA 3200 manufactured by the SLS [9]. A comparison of mechanical properties and surface texture of models produced by two AM technologies [10] was presented by Saharudin et al. Mesicek et al. [11] studied the use of the Abrasive surface finishing method for improving insufficient surface roughness of 3D-printed objects. An alternative way of smoothening the surface before 3D printing is described, e.g., by Bacciaglia et al. [12].

AM can help for example, in the manufacturing of proper cranial orthoses with predefined properties or of infills for helmets that will be light-weighted but strong enough to protect riders. The design of a protective helmet for riders using reverse engineering and AM was the brainchild of Wang and Co [13]. Using these techniques, a helmet was designed, the internal shape of which is based on the anatomical shape of the rider’s head. A cycling helmet that uses a Voronoi’s structure and has a better impact energy absorption is another example of such AM-produced protective devices. This approach, along with other uses of AM in the design of protective equipment, was introduced by Jefferson et al. [14] who produced a helmet made of Kevlar and fiberglass composite. In both cases, the possibility of good air circulation around the skin due to structured geometry is one of the key benefits of such helmets. Due to the suitable properties of flexible or general lattice structures, they are used in other industries as well. The design of lightweight parts with high strength and energy absorption capacity is used in the aircraft, rocket, automotive, and construction industries. Sharma et al. [15] dealt with the experimental and numerical study of a bioinspired structure as an energy absorber for automotive and aerospace industries. The methods of design and optimization of uniform lattice structures were dealt with by Pan et al. [16]. Numerical analysis of anisotropic lattices developed 25 years ago for spacecraft applications is presented by Vasiliev and Razin [17]. The design of lattice thermal insulators used in the construction industry was presented by Alqahtani et al. [18].

The use of lattice structure became an essential part of the design of prototype and production parts, offering a way to reduce the weight and thus the amount of material needed while maintaining high stiffness. These structures began to be used as the internal filling of components. Fernandez-Vicente et al. [19] presented the influence of lattice structure design and infill density on the tensile mechanical properties of such structures. Tao and Leu discuss AM processes, design methods and considerations, mechanical behavior, and applications for lattice structures using this technology [20]. The use of 3D printing in the production of biomedical or protective devices is on the rise, thanks to the possibility of high-speed production of parts and the ongoing research into new usable materials. Hazrat Ali et al. [21] presented finite element analysis of 3D-printed ankle-foot orthosis. The process of manufacturing a product by 3D printing can be described as the creation of a model placing layer by layer of material (depending on the chosen technology). 3D printing enables the possibility of creating a variety of complex shapes that would be difficult or impossible to create using the conventional methods mentioned above. The disadvantages of using 3D printing include the limitation of dimensions of the printed part (depending on the used printer), the need to create supports, and the minimum thickness of the part. For the production of the complex structures that are the subject of this paper, it is beneficial to use 3D printing technology that does not require supports. However, internal filling in the form of a lattice structure is not a suitable procedure to reduce skin contact. A suitable solution is to design the structure as flexible rather than unnaturally filled by the lattice structure from the beginning.

**Figure 1:**
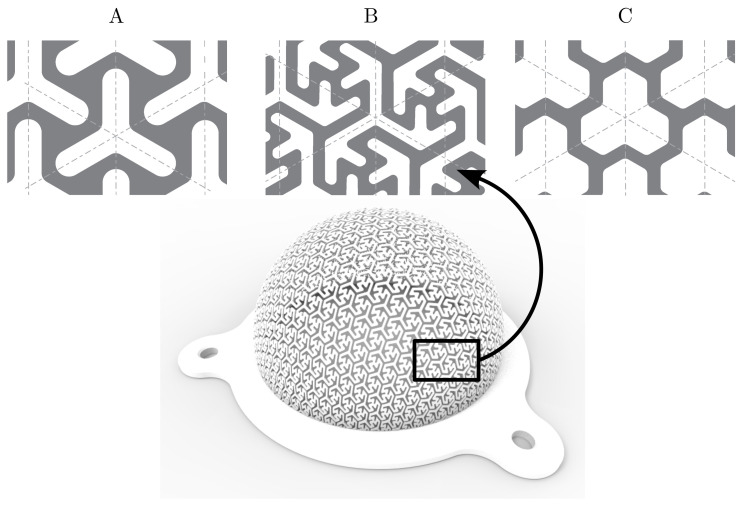
Flexible structure with three types of planar patterns presented in [26] (three-pointed star pattern (**A**), modified three-pointed star pattern (**B**) and tri-hexagonal pattern (**C**)).

Therefore, flexible structures (FS) are most commonly represents by thin shells formed of repeating identical units called cells. The main idea of such structures is to control their mechanical properties by changing the cell’s geometry rather than changing material to achieve the same properties. The design and study of structures with a given deformation behavior were reported in the paper [22] by Panetta et al. A method for designing shells with given structural integrity and aesthetic properties was presented by Schumacher et al. [23]. The same author also investigated the mechanical properties of structured sheet materials having direction-dependent macro mechanical behavior and aesthetic possibilities due to their appearance [24]. With the development of biocompatible materials for 3D printing, lattice structures have started to be used in biomedicine applications, such as implants. Kantaros and Piromalis [25] reviewed lattice scaffold structures for tissue regeneration. Based on all these studies, engineers can nowadays design unique structures customized for the user with respect to material savings controlled by the stiffness-mass ratio. The final mechanical properties of FS depend on the design and size of the individual cell. An example of a flexible structure sample with three types of planar patterns formed by identical cells is presented in Figure 1.

Marsalek et al. [26] prepared FS with planar patterns that were formed by repeating identical 2D cells. These patterns were projected onto curved surfaces and then swept to construct a 3D model, which can be printed using AM. These structures were 3D-printed using the SLS method and made of Nylon-Polyamide 12. The SLS method is based on layering the material in the form of powder and sintering it using a laser, the function of the supports being borne by the unsintered powder. Using experimental measurements, it was possible to determine the stiffness and load-bearing capacity (LBC) of five printed samples. The authors further showed the possibility of modelling the stiffness and LBC of this FS using a non-linear numerical model. However, the basic structure is formed by planar patterns that are stretched to create a 3D object. The proposed solution had either relatively high contact surface fraction (46 ÷ 56%) and low stiffness (4 ÷ 12 N/mm; pattern B in Figure 1), or high stiffness (48 ÷ 170 N/mm) and lower contact surface fraction (20 ÷ 24%; pattern C). Contact surface fraction is the ratio of the surface area of the printed pattern to the surface area of the entire curved surface. This property is particularly important to ensure good airflow between the structure and the skin, it also prevents sweating and itching of the skin and at the same time it has a significant effect on the amount of skin contact. Table 1 shows the stiffnesses of structures A, B, and C as well as the contact surface fraction values.

In the presented paper, the authors focused on the design of a new lightweight flexible structure (LFS). As this research builds on the paper by Marsalek et al. the same material and manufacturing technology are chosen to allow the assessment of the FS properties depending on its geometry and not on the choice of material. The aim was to develop a structure with contact surface fraction reduced to only 10% while preserving low stiffness and sufficient strength. Preliminary results of this work were published in Bachelor’s thesis [27] of Kusnir in 2021 and also presented at the international conference on Experimental Stress Analysis 2022 [28].

## 2. Materials and Methods

This work can be divided into 3 phases. In the first phase, the design of a lightweight flexible structure with a spatial pattern reducing contact surface fraction is described. In the next phase, laboratory testing of printed LFS samples is performed and in the third phase, results are used for the parametric study of pattern properties in a validated numerical model of LFS.

### 2.1. Design of the Spatial Pattern for the Lightweight Flexible Structure

**Figure 2:**
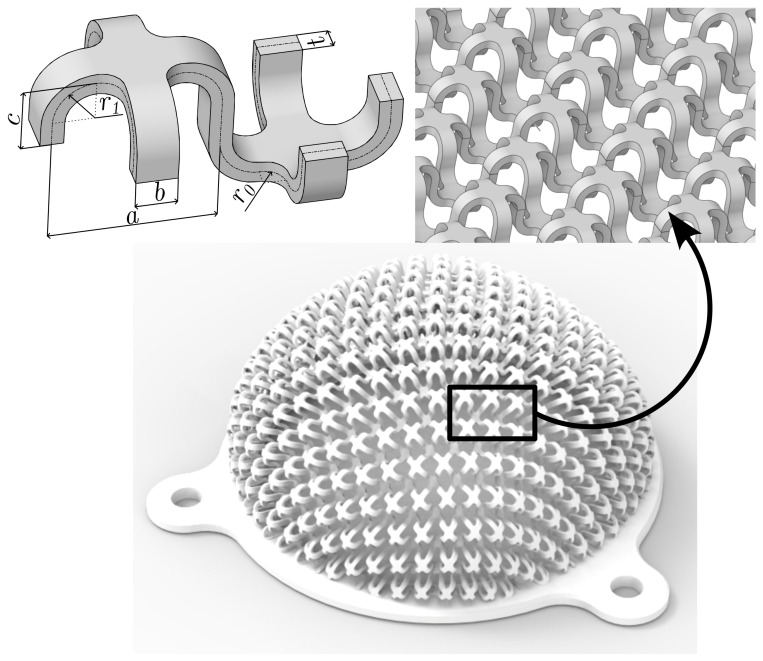
New lightweight spatial pattern design.

For the purpose of this work, it was necessary to design a periodic spatial repeating cell. The design of the cell was inspired by interconnected curved Swiss crosses. The individual parameters *a, b, c, $r_{0}$, $r_{1}$* of the designed pattern cell are shown in Figure 2 and dimensions are presented in Table 2.

This spatial pattern allows achieving a low contact surface fraction of only 10% for each of the variants. The parameter *t*, the thickness of the cell structure, investigated in this work ranged from 0.8–3.0 mm. A thickness of less than 0.8 mm resulted in poor quality printing of the samples, the upper limit of 3.0 mm is due to the loss of the initial cell shape.

The whole LFS was designed using a combination of two CAD SW, Rhinoceros (Robert McNeel & Associates, Seattle, WA, USA, RHN) and Inventor (Autodesk, San Francisco, CA, USA). In order to achieve the smallest possible deformation of the shape of the resulting LFS, it was first necessary to create a projection surface in Rhinoceros. The shape of the (projection) surface designed in Rhinoceros was close to hemispherical but not perfectly hemispherical, which resulted in significantly lower deformations of the resulting LFS than a perfectly hemispherical surface would have. In effect, the projected structure behaved according to our needs. The projection surface was subsequently unfolded into the plane, creating a rectangular surface that determined the dimensions for the preparation of the structure’s pattern. In Inventor, it was then possible to prepare the individual structures as planar plates, where the overall dimensions of the plate corresponded to a rectangle with a small overlap. This prepared surface could then be back-mapped onto the projection surface. This algorithm ensured the possibility of continuous mapping and resulted in an almost perfectly hemispherical structure. The resulting structure without a rim was then imported into Inventor, where this very complex and demanding STL model could be linked to the rim of the sample. The geometry of the specimen rim was designed to fit in a test stand (see [26]). This assembly of two parts was finally exported again as an STL model, thus creating a definitive blueprint for 3D printing.

**Figure 3:**
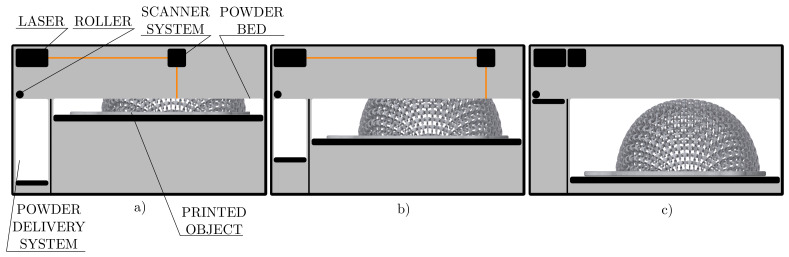
The scheme of SLS technology, (**a**) 25%, (**b**) 50%, and (**c**) 100% of LFS printing process.

To allow comparison with the previous study [26], the same 3D printing technology as in that paper (i.e., as for the same hemispherical structure created with planar patterns) was used, namely SLS technology, which allows printing without supports. An EOS P396 printer (Electro Optical Systems, Krailling, Germany) with a maximum product size of 340 × 340 × 620 mm was used for printing. The scheme of LFS printing process is shown in Figure 3.

A total of five samples with different parameters were printed, using the plastic material polyamide PA12 (or also referred to as PA2200). Initial material properties are taken from paper by Marsalek et al. [29] and listed in Table 3. This thermoplastic material is characterized by good chemical stability and can be used for biomedical applications. The effects of selected geometrical parameters on the elastic properties of this material were presented in [30].

Where *E* is Young’s modulus, μ is Poisson’s ratio, and ρ is density. $\sigma_{Y}$ is yield strength and $E_{T}$ is the tangent modulus; the latter two parameters are used in the numerical model to capture the plastic strain of LFS. Although the density is not used in the numerical simulation, it is possible to use it for determining the total density of the LFS.

### 2.2. Laboratory Testing of the 3D-Printed Lightweight Structure

**Figure 4:**
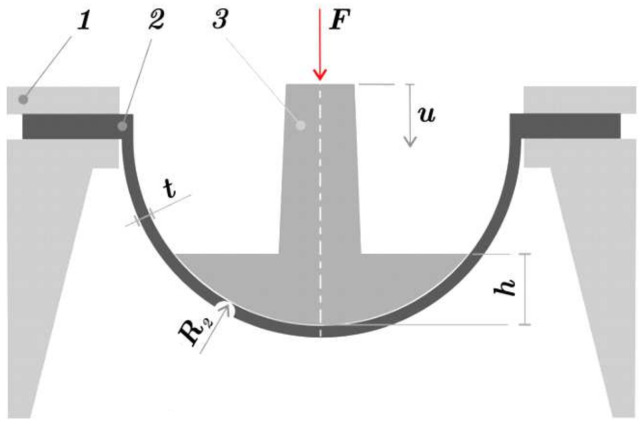
Scheme of experimental measuring used in [26].

The testing methodology of FS is based on previous research [26]. For the experiment, the following equipment was used: testing device (1), specimen (2), and plunger (3), the scheme of the testing assembly is shown in Figure 4. the plunger has a shape of a ball cap with a radius $R_{2}$ = 65 mm and height *h* = 18 mm, its surface is approximately 25% of the hemisphere’s surface. The whole assembly was put in the universal testing device TESTOMETRIC 500-50CT (Testometic, Rochdale, UK), the load was driven by displacement *u*, and the response force *F* was measured.

**Figure 5:**
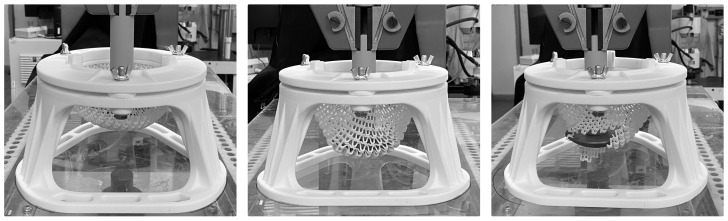
The process of laboratory testing of the 3D-printed lightweight structure stiffness. The initial setup (**left**), deformation of LFS during loading (**middle**) and damaged structure on reaching load-bearing capacity (**right**).

The specimen and testing device were 3D-printed using the SLS method from the PA12 material, the plunger was printed using the FDM (Fused Deposition Modelling) from polylactic acid (PLA). It must be emphasized that specimens were more compliant than the measurement device. The device was designed with robust legs with supports (see Figure 5). Also, both the fastening system and plunger were printed as full material (i.e., without using lattice infilling), which further increased their stiffness. The velocity of plunger displacement was set to 3 mm per minute and the specimen was loaded until the first cracks occurred. The process of laboratory testing of the 3D-printed LFS is on Figure 5.

### 2.3. Numerical Modelling of the Lightweight Structure

It is advisable to create a non-linear numerical model to reduce the cost of printing and experimental measurement. The advantages of these models over measurements include the speed of recalculation of new properties when the FS parameters are changed and, of course, null printing costs. The computational model was created in ANSYS Workbench 2021 R2 (Ansys, Canonsburg, PA, USA, AWB), employing the finite element method. The authors of this article have many years of experience in the application of numerical methods to solve the behavior of complex structures, such as numerical modelling of load-bearing capacity of steel-wire ropes by authors Hroncek et al. [31], modelling of impact test of rail wagon by Cech et al. [32], modelling of load-bearing capacity of single-stranded wire rope by Lesnak et al. [33], or modelling of cam design of spring-loaded camming device by Rybansky et al. [34].

**Figure 6:**
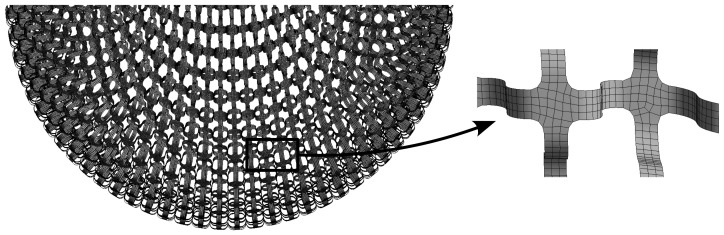
Finite element mesh with a detail of shell discretization.

The LFS itself is defined as shell midsurface, see Figure 2. To replace the continuum with finite elements, the Shell 181 element was used, with a thickness corresponding to t specific LFS. The discretization is shown in Figure 6. Each geometric model (I-III) has its own mesh, with approximately 211,000 nodes and 173,000 elements.

**Figure 7:**
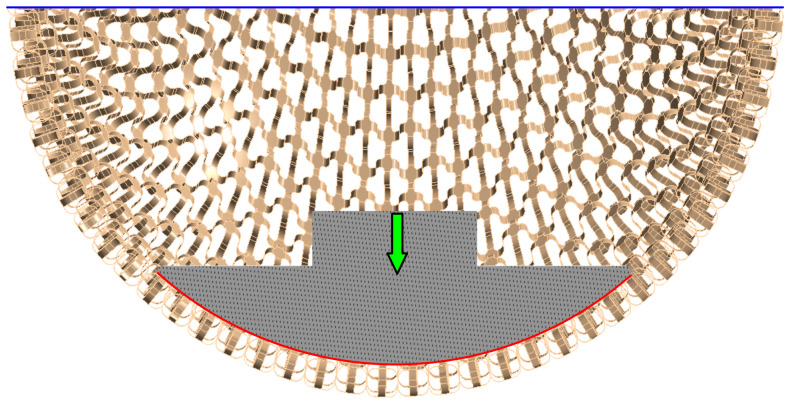
The scheme of the boundary conditions of LFS numerical model.

A non-linear numerical model was used to determine the deformation behavior, which consisted of a model of the LFS (without rim) and the geometry of the plunger. For the LFS, a bilinear elastoplastic material model with isotropic hardening was used, its parameters are given in Table 3. The plunger was modelled as rigid geometry because its stiffness is significantly higher than that of LFS. The boundary conditions were set to correspond most closely to the experimental ones, see Figure 7. The edge of the LFS was fixed (blue) and the load was applied using a plunger displacement function (green). A frictional contact between the LFS and the plunger was considered with a friction coefficient of $f_{s}$ = 0.30, same as in [26] (red). The computational model was considered with large deformations and displacements.

## 3. Results

**Figure 8:**
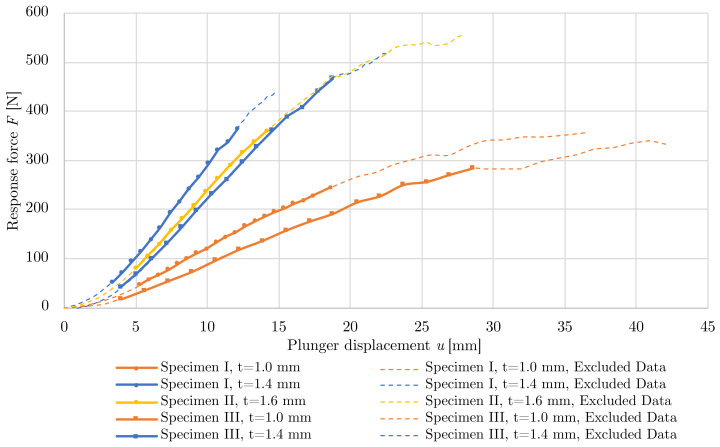
Force-displacement relationships for laboratory-tested specimens.

The laboratory testing was done for 5 printed samples. Figure 8 shows the relationships of the displacements on the reaction forces due to the LFSs deformations. Stiffness generally cannot be described as linear; however, after some initial deformation, the force-displacement diagram enters a linear phase. The initial non-linear deformation was caused by adjusting the contact between the specimen and the plunger, was different for each specimen, and was excluded from subsequent stiffness evaluation.

The stiffness of the structure is a fundamental design parameter for the design of a personalized LFS. The value of stiffness is defined as the slope of linear regression of non-excluded data so that the coefficient of determination is at least 0.99. LBC of the LFS in the load direction is also an important parameter, its values and evaluated stiffness are given in Table 4. Sample II with a thickness 1.60 mm achieved the highest LBC 554.00 N. The highest stiffness of 31.97 N/mm was achieved in sample I with a thickness 1.40 mm.

**Figure 9:**
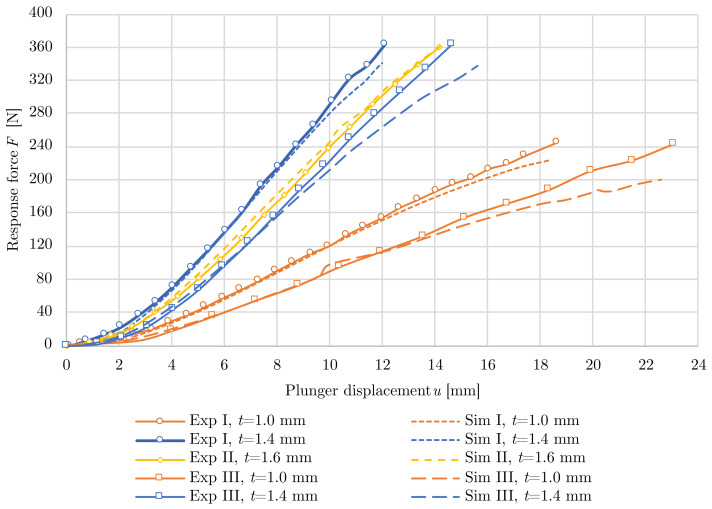
Comparison of the force-displacement relationships between experiment and simulation.

The presented non-linear numerical model was validated by measured data. The bilinear elastoplastic material model with isotropic hardening was employed. It took into account other non-linearities, such as large displacements and displacements and contacts. The results of the numerical model indicate a quality calibration with a maximum error between the model and the experiment of 4.00%, see Table 5. A comparison of the force-displacement diagram of the measurement and numerical models is shown in Figure 9.

**Figure 10:**
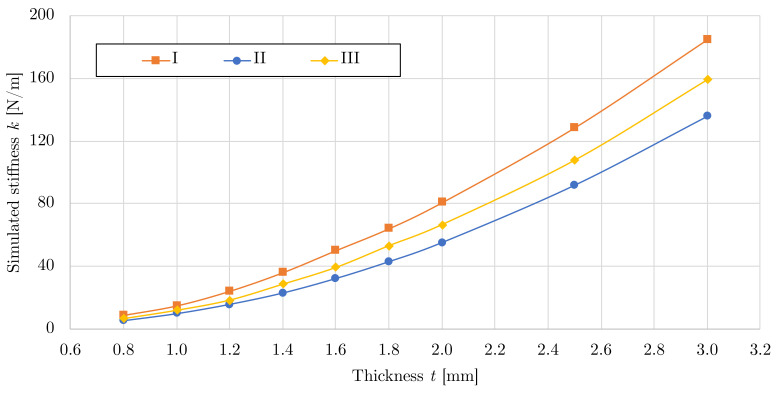
Results of a parametric study of the effect of LFS thickness on its stiffness.

With a prepared numerical model, a parametric study was performed to determine the effect of the thickness of the spatial structure on its stiffness, while a change in thickness has no influence on the contact surface fraction presented in Table 2. A total of 27 combinations of three LFS geometries with nine thicknesses were calculated, relationships between stiffness and thicknesses are shown in Figure 10 and summarized in Table 6. The computation time of one numerical model run s counted in hundreds of minutes on a standard machine (Intel i5-8600 workstation, 6 cores, 32 GB RAM). For example, the calculation time of the Variant III, *t* = 1.6 mm, was 515 min (443 iterations).

The maximum stiffness of 184.97 N/mm was achieved for model I with a thickness of 3.0 mm, while the lowest stiffness of 5.06 N/mm was achieved for model II with a thickness of 0.8 mm. The dependence of the stiffness on the simulated thickness is not linear, which is similar to the results of the LFS with a planar pattern [26], see Figure 10.

## 4. Discussion

The objective of this work was to design a lightweight flexible structure with spatial patterns. The stiffness of the designed LFS was evaluated using two methods, namely by experimental measurement and by numerical modelling using the Finite element method. Five printed samples were used for experimental testing. Experimental data were used for validation of the non-linear numerical model, which was used for the parametric study of the effect of thickness on the stiffness of LFS.

Although a parametric study of the effect of only thickness was performed, it would be advisable to study the effect of the other design parameters of the pattern, see Table 2. The resulting stiffness depends on the combination of all design parameters. It should be noted that modelled and simulated stiffnesses are validated in one loading direction only while in the other directions, the FS properties are likely to be different. Due to the relatively wide coverage of stiffness magnitudes, this research can be further used for the design of biomedical devices and help in deploying these FS (such as helmets, orthoses, prostheses, protective and healing equipment, etc.) in practice.

Although all parts of the assembly for experimental testing were 3D-printed, they were much stiffer than the tested specimen and, therefore, the flexibility of these parts was neglected. The testing device was also analyzed in a previous study using the finite element method [26], which proved how much deformation of the specimen was influenced by the compliance of these parts. The deformation of the testing device was negligible compared to the deformation of the spatial structure.

The stiffness characteristics are not linear during loading, but it is important to quantify the initial stiffness for the design of the LFS so that an adequate LFS can be matched to the purpose (orthoses, prostheses, etc.). When designing biomedical aids, it is necessary to design the LFS so that its range of applications lies primarily in the linear LFS loading response region.

In a previous study [26], FS with a planar pattern were presented. The disadvantage of these structures, however, was relatively large contact surface fraction at low stiffness. In the presented study, a reduction of contact surface fraction to approximately 10% was achieved while maintaining low stiffness (minimum 5.06 N/mm). The actual contact with the user’s skin will depend on the value of contact surface fraction, but also on other factors such as load, geometric design, selected material, loading on single cells, etc. The total thickness of the new spatial design (2·c+t, see Figure 2), is obviously greater than in the case of planar structures from the previous study. However, the new geometry has the advantage of reducing the possibility of skin scratching and clothing snagging caused by the sharp geometry of the planar design. The geometry printed using the SLS method shows a high surface roughness, which could be reduced by using the vaporization smoothing method [35]. This technique closes the external pores on the surface and allows improvement of the mechanical properties of the printed parts [36].

Numerical models include all types of non-linearities (non-linear material model, large deformations, and contacts). This relatively complex simulation can be more accurate using a sophisticated material model (the bilinear model with isotropic hardening is acutely used). The model also does not include the effect of possible anisotropic material behavior due to the direction during printing. For the numerical simulation, a relatively high computational performance is required for this primary study. For the design of real equipment, the geometry of the task, different loading states, etc. would increase this complexity.

Load-bearing capacity (LBC) was also experimentally determined as the load at which the structure was damaged. The fact that LBC was not numerically modelled can be considered a limitation of the presented study. However, developing such additional computations is beyond the scope of the presented study and as it is a complicated process, would inflate the length of the paper over a reasonable level. Another possible area of study is the modelling of load-bearing capacity of individual cells and their deformation behavior. To determine whether a single cell can be turned inside out without destroying, a numerical analysis of the LFS (Variant II) with 0.8 mm thickness was performed. During loading, the limit plastic strain was exceeded before the cell was turned inside out. The limit plastic strain is based on a previous study result [26]. It should be also noted that in real life, other parameters, such as fatigue properties, play an important role in the usability of the designed structures, which should also be a subject of future studies.

## 5. Conclusions

In this work, a lightweight flexible structure, the stiffness of which is variable depending on the final application, which also offers a greatly reduced contact surface fraction to as low as 10%, was designed. The newly designed spatial LFS used in biomedical applications might provide better sweat drainage, airflow, and lower skin contact, which could increase the patient’s comfort. Using two methods (experimental and numerical approaches), 5 printed samples were compared. Non-linear computer modelling of such a pattern has proved to be a useful tool, yielding results highly consistent (<4% difference) with the experimental evaluations. Although experimental stiffness determination is a more accurate tool, numerical modelling is particularly advantageous in the case of parametric studies.

Using a parametric model validated using experimental results, a total of 36 specimens with stiffness ranging from 5.06–184.97 N/mm were produced. The variant II with a thickness of 0.8 mm was determined as the LFS variant with the lowest stiffness of 5.06 N/mm. This pattern variant provides consistently the lowest stiffness at each modelled thickness compared to other patterns, see Figure 10. As can be seen from this figure, the relationship of stiffness on modelled thickness is non-linear in the defined region, unlike the previous study with 2D flexible structures, where the relationship is linear.

## Figures and Tables

**Table 1:** Stiffness and contact surface fraction of individual pattern shown in Figure 1.

Pattern	Stiffness [N/mm]	Thickness [mm]	Contact Surface Fraction [−]
A	57.52 ÷ 208.22		44.4% ± 3%
B	3.98 ÷ 12.46	0.80 ÷ 2.05	51.4% ± 5%
C	47.72 ÷ 169.74		22.4% ± 2%

**Table 2:** Parameters of the designed pattern cell.

Variant	*a* [mm]	*b* [mm]	*c* [mm]	$r_{0}$ [mm]	$r_{1}$ [mm]	Contact Surface Fraction [−]
I	7.4	1.8	2.5	0.9	1.9	
II	8.0	2.0	3.2	1.0	2.0	10% ± 1%
III	8.6	2.2	3.0	1.1	2.1	

**Table 3:** Mechanical properties of PA12 presented in [29].

Symbol	*E* **[MPa]**	μ [−]	ρ **[kg m** ${}^{-3}$ **]**	$\sigma_{Y}$ **[MPa]**	$E_{T}$ **[MPa]**
Value	1224	0.39	1010	21	334

**Table 4:** Measured stiffness *k*.

Specimen	Thickness [mm]	Measured Stiffness [N/mm]	Load Bearing Capacity [N]
I	1.40 1.00	36.82 15.06	450 357
II	1.60	31.97	554
III	1.40 1.00	29.04 11.25	520 343

**Table 5:** Comparison of the stiffness *k* between experiment and simulation.

Specimen	Thickness [mm]	Measured Stiffness **[N/mm]**	Simulated Stiffness **[N/mm]**	Stiffness Difference **[%]**
I	1.40 1.00	36.82 15.06	35.89 14.68	2.53 2.52
II	1.60	31.97	32.05	0.25
III	1.40 1.00	29.04 11.25	28.38 11.7	2.27 4.00

**Table 6:** Relationships between simulated thickness and stiffness *k* [N/mm] of LFS.

Variant	Simulated Thickness *t* [mm]								
	3.0	2.5	2.0	1.8	1.6	1.4	1.2	1.0	0.8
I	184.97	128.56	80.65	64.00	49.99	35.89	24.03	14.68	8.61
II	135.79	91.72	55.06	42.84	32.05	22.79	15.51	9.56	5.06
III	159.02	107.60	66.45	52.98	39.09	28.38	18.22	11.70	6.38

## Data Availability

Data sharing is not applicable to this article.

## Abbreviations

The following abbreviations are used in this manuscript:

2D	2-Dimensional
3D	3-Dimensional
AM	Additive Manufacturing
AWB	Ansys Workbench 2021R2
CA	California
CAD	Computer-Aided Design
FDM	Fused Deposition Modeling
FS	Flexible Structure
LBC	Load-Bearing Capacity
LFS	Lightweight Flexible Structure
PLA	Polylactil Acid
PA12	Polyamide 12
RAM	Random-Access Memory
SLS	Selective Laser Sintering
STL	Standard Triangle Language
UK	United Kingdom
USA	United States of America
WA	Washington
